# Breast cancer tumour growth modelling for studying the association of body size with tumour growth rate and symptomatic detection using case-control data

**DOI:** 10.1186/s13058-015-0614-z

**Published:** 2015-08-21

**Authors:** Linda Abrahamsson, Kamila Czene, Per Hall, Keith Humphreys

**Affiliations:** Department of Medical Epidemiology and Biostatistics, Karolinska Institutet, Box 281, Stockholm, SE-171 77 Sweden

## Abstract

**Introduction:**

A large body size is associated with larger breast cancer tumours at diagnosis. Standard regression models for tumour size at diagnosis are not sufficient for unravelling the mechanisms behind the association.

**Methods:**

Using Swedish case-control data, we identified 1352 postmenopausal women with incident invasive breast cancer diagnosed between 1993 and 1995. We used a novel continuous tumour growth model, which models tumour sizes at diagnosis through three submodels: for tumour growth, time to symptomatic detection, and screening sensitivity. Tumour size at other time points is thought of as a latent variable.

**Results:**

We quantified the relationship between body size with tumour growth and time to symptomatic detection. High body mass index and large breast size are, respectively, significantly associated with fast tumour growth rate and delayed time to symptomatic detection (combined *P* value = 5.0 × 10^−5^ and individual *P* values = 0.089 and 0.022). We also quantified the role of mammographic density in screening sensitivity.

**Conclusions:**

The times at which tumours will be symptomatically detected may vary substantially between women with different breast sizes. The proposed tumour growth model represents a novel and useful approach for quantifying the effects of breast cancer risk factors on tumour growth and detection.

## Introduction

Among postmenopausal women, a high body mass index (BMI) is known to be associated with an increased risk for breast cancer [1]. High BMI has also been shown to be associated with large tumour size [2–4]. These associations have been reported on the basis of fitting standard regression models to large data sets. The association between BMI and tumour size could be due to a large body size being associated with fast tumour growth or a delayed symptomatic detection or both.

To evaluate the full impact of body size on tumour growth/symptomatic detection, it is important to incorporate mammographic density at the analysis stage. Mammographic density refers to the tissue composition of a woman’s breast as seen on a mammogram. Fibro-glandular tissue is radiodense and appears white on a mammogram, whereas fat is radiolucent and appears dark on a mammogram. Body size, as measured by BMI, is negatively correlated to percentage mammographic density (PMD) (the percentage of the breast area appearing ‘dense’ on a mammogram), which in turn is associated with larger tumour size because of lower screening sensitivity [5–7].

The hypothesis that BMI is associated with tumour growth is supported by studies with molecular markers. In a retrospective cohort study of women enrolled in a screening programme in western Washington state, it was reported that obese women had significantly faster-growing tumours, as measured by Ki-67 [8]. High BMI is considered to be linked to fast tumour growth through locally increased estrogen levels [9–11]. However, as we have already suggested, tumour growth rates may not represent the only mechanism through which body size and tumour size at diagnosis are linked. A difficulty to find tumours symptomatically in large breasts has also been hypothesised to contribute to the association between BMI and tumour size at diagnosis [2–4].

**Fig. 1:**
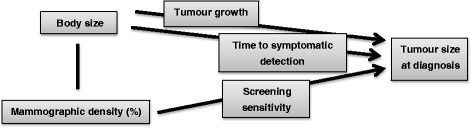
Relationships between body size and mammographic density and breast cancer tumour size at diagnosis

Although standard regression techniques can be used to evaluate (overall) associations between tumour size and body size covariates, more sophisticated approaches are needed to shed light on the mechanisms underlying these associations. In this article, we quantify the relationships between body size covariates (BMI and breast size) and tumour growth/symptomatic detection as well as the role of mammographic density in screening sensitivity (Fig. 1). We do this by using epidemiological data and an extension of a novel statistical modelling approach recently described by our research group [12]. Since tumours in pre- and postmenopausal women grow with different speeds [13], we restrict our analysis to postmenopausal women. Within our approach, three submodels are specified: for tumour growth, time to symptomatic detection, and mammography screening sensitivity. Because information on tumour size is available only at the time of detection (at least in our study), we treat it as a latent variable at other time points. Underlying processes assumed in the submodels, together with information on each woman’s screening history and detection mode, are then used to find probabilities of different tumour sizes at time points of negative screenings and hypothetical times of symptomatic detection (for screening cases).

The extension of the model that we present in this work makes it possible to include covariates in all submodels, enabling us to jointly evaluate how particular factors, such as body size, affect tumour growth, symptomatic detection, and screening sensitivity. To the best of our knowledge, ours is the first approach to enable this.

## Methods

### Data

The study population consists of postmenopausal women born in Sweden with a primary invasive breast cancer. It originates from the cases in a case-control study of women who were between 50 and 74 years old and whose cancer was diagnosed between 1 October 1993 to 31 March 1995 [14]. Through Swedish Regional Cancer Registries, 3979 women were identified as being a case and thus invited to the study. From 66 out of the 68 Swedish mammography screening units and radiology departments, information regarding dates and reasons for mammographies (screening or referral) performed within 3 months to 5 years before diagnosis (excluding the occasions closest in time to diagnosis to avoid registering diagnostic examinations) was collected [15]. Also, mammographic images (mediolateral-oblique view) were gathered, and these have been digitised for most of the participating women. From the images, PMD and total breast area (TBA) in pixels were estimated during 2007 and 2008 [6] by a trained user of the computer programme Cumulus. Both tumours and dense tissues are bright on mammograms and therefore the PMD and TBA estimations were made on the most recent mammograms of the contralateral breast before diagnosis in order to avoid overestimating PMD.

Information about tumour size and other tumour characteristics, as well as neoadjuvant treatment and reason for diagnostic mammography, was retrieved from surgical and oncological patient records throughout Sweden. Through self-reported questionnaires, we collected information about breast cancer risk factors, such as height and weight.

Of the 3979 women identified as cases, 84 % (3345) chose to participate. However, 320 participants were excluded because of a noninvasive breast cancer diagnosis, a previous or other cancer diagnosis, or the lack of written consent or because their diagnosis was made before or after the study period. Furthermore, 177 premenopausal women were excluded, as were 168 women with unknown age at menopause. Screening history was missing on 626 cases, so these women were also excluded, as were 50 cases with unknown detection mode (i.e., being found through screening or symptomatically). Also, 24 women had no information on tumour size, and 611 women were lacking available mammograms and therefore were also excluded here. Six cases had no information on BMI, calculated from height and weight, and 11 women received neoadjuvant treatment before the pathologic measurement of the tumour size and therefore were excluded. From the original case-control study, 1352 women are included in the analyses here; 937 were screening cases and 415 were symptomatic cases. Of the symptomatic cases, 292 women (70 %) had a tumour diagnosed within 2 years of a negative screening. Prior to the exclusion of women without screening history or available mammograms, data for 2680 women were available for analysis. The age and tumour size (diameter) distributions—25th, 50th, 75th percentiles of (58, 64, 69) for age (years) and (10, 15, 22) for tumour size (millimetres)—in this group are similar to those in the group of women included in the analysis presented in this article: (58, 63, 68) for age (years) and (10, 15, 20) for tumour size (millimetres). Ethical approval for the study was obtained from the Regional Ethics Review Board in Uppsala at Uppsala University (reference number 155/93), and ethical approvals of extensions of the study were obtained from the Regional Ethics Review Board in Stockholm at Karolinska Institutet (reference numbers 98-226 and 99-338). All participants provided written informed consent.

### Statistical analysis

We first carried out an explanatory data analysis, tabulating relationships between the key variables tumour size, BMI, and TBA. Subsequent analyses were based on our tumour growth model, which we describe below.

Our main approach is based on modelling the size of a woman’s tumour (at detection), conditioning, explicitly, on her screening history (dates of previous mammography screens), and how her tumour was detected (screening/symptomatic detection). We also allow tumour size (at detection) to be dependent on body size and PMD. This is done by specifying and using three submodels (described below) for tumour growth, time to symptomatic detection, and screening sensitivity (as functions of body size covariates and PMD). Although calculations are complex, only eight parameters are estimated. The unknown parameters are estimated by optimising a likelihood function. The model described in this article is based on a non-trivial extension of the approach of Abrahamsson and Humphreys [12] (which did not allow covariates for tumour growth and time to symptomatic detection) so that submodels can be functions of covariates, such as body size and PMD. Details and formulas of the model and its extensions are given in the statistical methods Appendix.

We decided to use BMI as a body size covariate in the submodel for tumour growth since it is believed to have a close link to estrogen levels and therefore to tumour growth rate. For the submodel of symptomatic detection, TBA was used as a proxy for breast size, which has a closer relation to detection, through palpation, than BMI. Although we use different covariates for body size in these two submodels, they have a strong positive correlation (Spearman’s rank correlation coefficient of 0.65), which will diminish their individual associations with tumour size. This has been shown for standard regression models in a study of clinical stage with BMI and breast size as covariates [16]. In the submodel for screening sensitivity, PMD was used as proxy for mammographic density.

### Submodels

The first submodel is for tumour growth. Tumours are assumed to be spherical and to grow exponentially with a constant volume doubling time. To allow for tumours to grow at different rates, the growth rate is modelled as a function of BMI (centered on the mean value in the study) through the parameter *α*_2_. There are two other parameters, *α*_1_ and *σ* ^2^, which are used to specify the general form of the tumour growth function (see the Appendix). The tumour growth model, which has been chosen partly because of mathematical tractability, has also been used by Bartoszyński et al. [17] and Plevritis et al. [18]. Neither of those articles allowed growth rates to vary according to an observed factor/covariate. The second submodel is for time to symptomatic detection. We assume that time to symptomatic detection depends on the size of the tumour through a hazard function, as is also done in Bartoszyński et al. [17] and Plevritis et al. [18]. Unlike in previous work, we allow time to symptomatic detection to depend on breast size, as measured by the standardised TBA (centered on its mean value and divided by its standard deviation) calculated from mammograms. Two parameters, *η*_1_ and *η*_2_, are included in the submodel for time to symptomatic detection. Inference on *η*_2_ provides information on the relationship between the size of a woman’s breast and time to symptomatic detection (caused by difficulties in palpation). The parameter *η*_1_ is for the general form of the hazard function. The third submodel is for the screening sensitivity. Larger tumours are easier to find through screening than small ones, and tumours can also be masked in breasts with high mammographic density. We therefore assume mammography screening sensitivity (which is the probability that, from a mammographic image, a radiologist will detect a tumour) to be a function of tumour size and mammographic density. Its functional form is adopted from Weedon-Fekjær et al. [19]. Three parameters—*β*_1_, *β*_2_, and *β*_3_—are required to be estimated. *β*_2_ links tumour size (diameter in millimetres) to screening sensitivity, and *β*_3_ links PMD to screening sensitivity.

### Parameter estimation

The values of the parameters *α*_1_, *α*_2_, *σ* ^2^, *η*_1_, *η*_2_, *β*_1_, *β*_2_, and *β*_3_ are estimated by maximising a likelihood function, which is carried out by using the modified quasi-Newton optimisation procedure called L-BFGS-B in the optim function in the statistical programme *R* [20]. Calculation of the likelihood takes some time because it is based on summing different quantities over a number of possible categories of tumour sizes (at symptomatic detection) and time lags. To speed up parameter estimation, we divided BMI (and TBA) values into 10 small categories, labelling each category with its midpoint. In this way, parts of the likelihood functions could be evaluated for several women at once. As in our earlier study [12], a maximum number of three earlier negative screenings was used in the calculations to ease computations. Tumour size in diameter, at the time point of detection, was categorised into the following intervals (in millimetres): [0,1.5), [1.5,2.5), [2.5,7.5), [7.5,12.5), [12.5,17.5),…, [67.5,72.5), [72.5,85), [85,95), [95,105),…, [145, 155]. This is important since some pathologists tend to round off tumour size values to the nearest 5 or 10 mm, especially for large tumours.

To obtain estimates of variability for the point estimates, we used the profile likelihood function to calculate 95 % confidence intervals. Likelihood ratio tests were carried out to assess whether BMI, TBA, and PMD were associated with growth, symptomatic detection, and screening sensitivity, respectively. A non-parametric bootstrap approach [12] and the percentile method for 100 bootstrap replications was used to plot 95 % confidence regions for the screening sensitivity estimates.

## Results

Key characteristics of the data are presented in Table 1. Prior to applying our tumour growth model to the data, we calculated the median sizes of tumours within groups defined by BMI and TBA, for screening and symptomatically diagnosed cases, separately (Table 2). Aside from symptomatically detected tumours being consistently (much) larger than screening detected tumours, the most marked differences in tumour sizes (3.5 to 4 mm) were, for symptomatically detected tumours, between TBA groups, suggesting that TBA plays a role in delaying time to symptomatic detection. High BMI values were associated with slightly larger, or roughly equal-sized, tumours (0 to 2 mm differences) within groups defined by detection mode and TBA. Given the results of this preliminary analysis, it seemed sensible to proceed with fitting our tumour growth model in order to quantify the relationships between TBA and time to symptomatic detection and between BMI and tumour growth rate.

**Table 1:** Descriptive comparison of screening and symptomatically detected cancers

	Detection mode		
	Screening	Symptomatic with a negative screen in the last 2 years	Symptomatic without a negative screen in the last 2 years
Number of cases	937	292	123
Tumour size, mm	12.00	19.00	20.00
(median and quartiles)	(9.00, 18.00)	(13.75, 25.00)	(13.50, 28.00)
Age at diagnosis	63	62	65
(median and quartiles)	(58, 68)	(56.75, 67)	(61,71)
Body mass index	25.65	24.78	24.45
(median and quartiles)	(23.23, 28.26)	(22.31, 27.65)	(22.71, 27.19)
Percentage mammographic density	13.51	15.71	14.77
(median and quartiles)	(6.33, 23.72)	(7.90, 29.44)	(9.04, 28.83)
Standardised total breast area	0.05	−0.32	−0.15
(median and quartiles)	(−0.67, 0.74)	(−1.04, 0.40)	(−0.73, 0.74)
Cases with no previous negative screening	17	0	13
Cases with 1 previous negative screening	200	22	57
Cases with 2 previous negative screenings	596	149	49
Cases with 3 previous negative screenings	124	121	4
Time since last negative screening, years^a^	2.01	1.19	2.67
(median and quartiles)	(1.79, 2.13)	(0.90, 1.57)	(2.24, 3.56)

^a^For cases with at least one previous negative screening

**Table 2:** Tumour diameters for symptomatically and screen-detected cancers

Symptomatically diagnosed cases:				
	Standardised total breast area ≤0		Standardised total breast area >0	
	Body mass index ≤25	Body mass index >25	Body mass index ≤25	Body mass index >25
Tumour size	17.00 (12.00, 23.00)	18.50 (12.00, 24.75)	21.00 (15.00, 25.75)	22.00 (15.00, 30.00)
	*n* = 191	*n* = 58	*n* = 34	*n* = 132
Screening diagnosed cases:				
	Standardised total breast area ≤0		Standardised total breast area >0	
	Body mass index ≤25	Body mass index >25	Body mass index ≤25	Body mass index >25
Tumour size	12.00 (10.00, 18.00)	12.00 (8.00, 17.00)	11.00 (8.00, 19.00)	13.00 (9.00, 19.50)
	*n* = 308	*n* = 145	*n* = 105	*n* = 379

Tumour diameters in millimetres (median and quartiles) grouped by body mass index and standardised total breast area

**Fig. 2:**
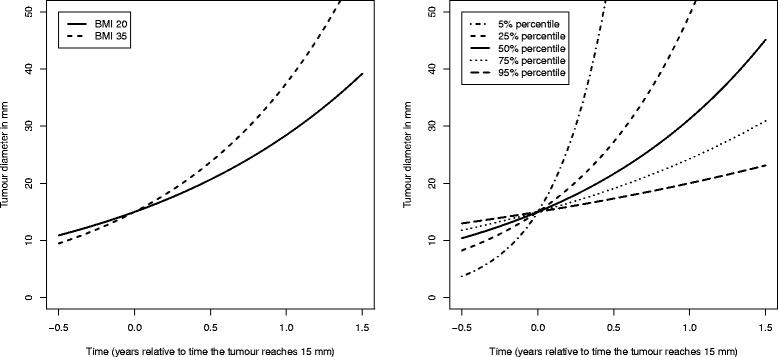
Tumour growth functions. (Left) Tumour growth functions for the estimated median inverse growth rate for women with BMI 20 and 35. (Right) Tumour growth functions (at 5, 25, 50, 75 and 95 percentiles for the estimated inverse growth rate) for women with mean BMI (25.71)

Parameter estimates (point estimates and 95 % confidence intervals from the profile likelihood) for the tumour growth model described in the Methods section are displayed in Table 3. We fitted a number of nested models in order to carry out likelihood ratio tests for key parameters (Table 4). The *P* value associated with adding both BMI and TBA to the model was small (5.0 × 10^−5^). The *P* value from testing the (individual) association between BMI and tumour growth was 0.089 (likelihood ratio test), whereas the *P* value for TBA and time to symptomatic detection was 0.022. The main purpose of our tumour growth model analysis is to learn about the direct role of BMI/TBA in tumour growth/time to symptomatic detection. In Fig. 2, we plot estimated tumour growth based on our sample of 1352 women with breast cancer. Estimated tumour growth curves are plotted (left of Fig. 2) for women with BMIs of 20 and 35. As in Weedon-Fekjær et al. [19], time is plotted on the x-axis and time point 0 represents the time at which the tumour had a diameter of 15 mm. Tumours in lean women are estimated to have a slower growth than tumours in heavy women. The estimated variation in growth rates is represented in the plot to the right in Fig. 2.

**Table 3:** Parameter estimates of tumour growth, time to symptomatic detection, and screening sensitivity

Model for tumour growth:		
Parameter	Point estimate	Confidence interval (profile likelihood)
Intercept, *α*_1_	−0.65	(−0.99, −0.28)
Body mass index coefficient, *α*_2_	−0.02	(−0.05, 0.00)
Coefficient of variation, *σ* ^2^	0.40	(0.31, 0.52)
Model for screening sensitivity:		
Parameter	Point estimate	Confidence interval (profile likelihood)
Intercept, *β*_1_	−4.68	(−5.10, −4.30)
Tumour size coefficient, *β*_2_	0.57	(0.49, 0.66)
Percentage mammographic density coefficient, *β*_3_	−2.39	(−3.81, −1.01)
Model for time to symptomatic detection:		
Parameter	Point estimate	Confidence interval (profile likelihood)
Intercept, *η*_1_	−8.26	(−8.82, −7.88)
Total breast area coefficient, *η*_2_	−0.17	(−0.32, −0.04)

Parameter estimates are presented with 95 % confidence intervals

**Table 4:** *P* values from likelihood ratio tests

Null hypothesis	Corresponding covariate (submodel)	*P* value	Log-likelihood
*α*_2_ = 0, *η*_2_ = 0	BMI (tumour growth) and TBA (time to symptomatic detection)	5.0 × 10^−5^	−2597.790
*α*_2_ = 0	BMI (tumour growth)	0.089	−2589.324
*η*_2_ = 0	TBA (time to symptomatic detection)	0.022	−2590.493
*β*_3_ = 0	PMD (screening sensitivity)	6.3 × 10^−4^	−2593.724

*P* values together with log-likelihood function values for covariates in models for tumour growth, time to symptomatic detection, and screening sensitivity. The log-likelihood function value for the full model is −2587.879

*BMI* body mass index, *TBA* total breast area, *PMD* percentage mammographic density

**Fig. 3:**
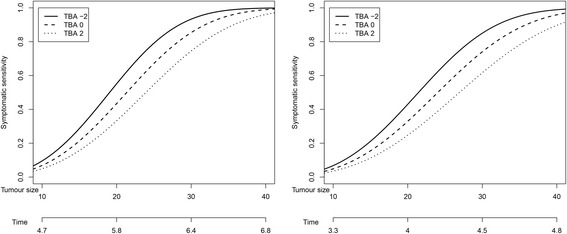
Estimated symptomatic sensitivities for women with small and large breasts. The estimates are based on the tumour growth function for the estimated median inverse tumour growth rate of a woman with BMI 20 (left) and BMI 35 (right). Time is counted, in years, from the point at which the tumour was 0.5 mm

**Fig. 4:**
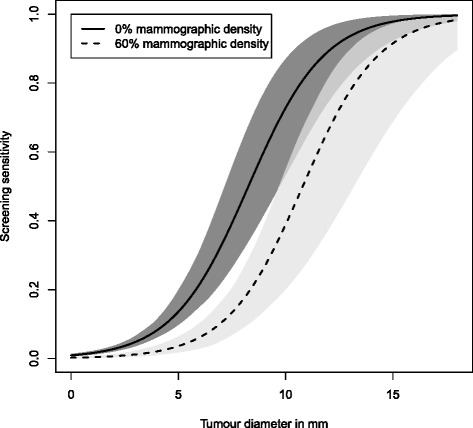
Estimated sensitivity functions with 95 % confidence intervals

Figure 3 represents the probability that a tumour, in the absence of screening, will have been symptomatically detected at a particular time point (which we refer to as symptomatic sensitivity) as a function of tumour size and size of the breast. Time is measured from the point when the tumour diameter was 0.5 mm. Symptomatic detection is estimated to take longer in larger breasts. For example, in a woman with a BMI of 20, we estimate that by the time a tumour has reached a diameter of 20 mm, in a woman with small breasts (two standard deviations below the mean value), the tumour will have been symptomatically detected with a probability of 0.55, whereas for a woman with large breasts (two standard deviations above the mean value) the corresponding probability is 0.33. The same probabilities in a woman with a BMI of 35 are, respectively, 0.43 and 0.24. In Fig. 4, estimates for screening sensitivity as a function of tumour size are plotted for women with 0 % and 60 % PMD. As expected, screening sensitivity is higher for women with less dense breasts.

**Fig. 5:**
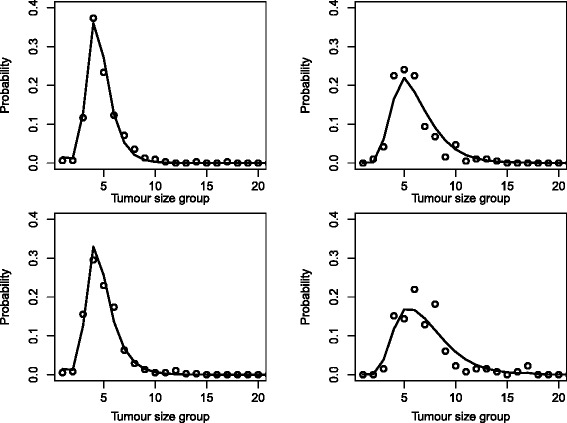
Fit of the tumour growth model. Fit of the model for screening detected cases (*left*) and symptomatic cases (*right*), divided into women with small body size (*top*; BMI of not more than 25 and TBA of not more than 0) and large body size (*bottom*; BMI of more than 25 and TBA of more than 0). Observed tumour size proportions are represented as dots and fitted proportions are represented as lines. *BMI* body mass index, *TBA* total breast area

To assess model fit, we graphically compared observed and fitted tumour size group proportions, the latter being calculated by summing fitted, individual probabilities. We did this within groups of women defined according to body size and detection mode so that we could also visualise differences between fitted tumour size distributions according to these factors (Fig. 5). The “larger” body size group was defined as having a BMI of more than 25 and a standardised TBA of more than 0 (511 women; Table 2), whereas the “smaller” body size group was defined as having a BMI of not more than 25 and standardised TBA of not more than 0 (499 women; Table 2). The model fits reasonably close to the observed tumour size distribution and is able to capture that the women with larger body sizes have, on average, larger tumours.

## Discussion

In this study, we have quantified the relationship between body size covariates (BMI and TBA, respectively) with tumour growth and time to symptomatic detection, as well as the role of PMD in screening sensitivity, using a novel continuous tumour growth model. Owing to the extension of the model that we present, this is the first time that breast cancer risk factors have been included in all parts of a statistical growth model which jointly studies tumour growth, screening sensitivity, and time to symptomatic detection. Only a few earlier studies have included risk factors in statistical tumour growth models. In a study with a multi-state model setting, Chiu et al. [21] estimated screening sensitivity, taking mammographic density into account. Our research group [12] has earlier estimated screening sensitivity as a function of PMD in a continuous tumour growth model without modelling systematic differences in tumour growth rates. Tumour growth and screening sensitivities were studied in two different age groups (50–59 and 60–69 years old) by a Norwegian group [19]. The authors found that younger women had faster-growing tumours. In a cohort study of Taiwanese women with simulated risk factors, Wu et al. [22] used a three-state Markov model setting (free of breast cancer, preclinical screen-detectable phase and clinical phase) and included risk factors, testing whether they were initiators or promoters (e.g., involved in tumour progression) of breast cancer. However, their simulated risk factors do not fully resemble real data and the test procedure takes into account only whether risk factors are more common in screening or clinical cancers.

Before carrying out our analysis, we checked/evaluated our modelling procedure by carrying out a simulation study. Simulations were performed in order to ensure that our computer programme was able to retrieve “true” parameter values. The simulations were carried out by using an approach similar to the one described by us before [12], but this time we included a single covariate in the submodels for tumour growth or time to symptomatic detection (the same covariate in the two submodels) or both. We did this to check model identifiability. We found that, as long as the covariate was included in both submodels (tumour growth and the time to symptomatic detection), estimation was valid. When the model was misspecified, parameter estimates were misleading; for example, when we generated data from a model where the covariate influences only tumour growth and then fitted a model where the covariate was excluded from the model for tumour growth but was still included in the model for time to symptomatic detection, the model estimated that the covariate was associated with time to symptomatic detection. This happens because late symptomatic detection and fast tumour growth are both associated with larger tumours at diagnosis. Although parameter identifiability is not a problem per se, this simulation underscores the importance of specifying a realistic model. In our case, we postulated that BMI was the most relevant covariate to use for tumour growth but that TBA was most relevant for time to symptomatic detection. Difficulties caused by related variables playing roles in both tumour growth and detection are not unique to our approach; this issue is essentially the same as that mentioned in the Methods section and discussed in a study using standard regression [16]. The advantage of our approach, over standard regression, is that it can provide insight and direct estimates of the role of factors (such as BMI, TBA, and PMD) in tumour growth, symptomatic detection, and screening sensitivity. Realistic models, however, need to be specified.

The estimates in this study regarding the overall tumour growth and the overall symptomatic sensitivity (the overall probability that a tumour has been symptomatically detected through palpation, by a specific time point, in the absence of screening) were similar to the results in our earlier study [12]. There are no previous studies estimating the dependence of tumour growth on BMI and the dependence of symptomatic sensitivity on TBA to which we can compare our results. Our estimate of screening sensitivity, as a function of tumour size and PMD, also resembles our earlier estimate [12].

In this work, we assume an exponential growth model with a gamma distribution for the inverse tumour growth rate. This made it possible to make the extension of including covariates in the submodels. Analytically tractable solutions would not be possible within a likelihood framework if other models, such as logistic growth, were used. However, in Fig. 5, we see that, although among symptomatically detected cancers there may be a tendency for our model to slightly overestimate the number of moderately large tumours, overall the model provides a good fit to the data.

From Fig. 3, it can be seen that symptomatic sensitivity approaches 1 when tumours are around 40 mm. Although 40 mm might seem large, tumours are detected symptomatically at such sizes in the absence of screening [18].

With BMI and TBA included in our tumour growth model, our point estimate of the effect of PMD on screening sensitivity was −2.39 (*P* value = 6.3 × 10^−4^; Tables 3 and 4) corresponding to a per-standard deviation odds ratio of 0.71. We note that when BMI and TBA were not included, we obtained a point estimate of −1.96 (*P* value = 4.0 × 10^−3^) and a per-standard deviation odds ratio of 0.75. These estimates differ presumably because of the negative correlation between PMD and the other variables and underscore the importance of specifying each submodel as well as possible.

Although it is clear that including both BMI and TBA significantly improves the fit of our tumour growth model compared with a model with neither of these factors, we were not able to show that, when TBA is included in the model for symptomatic detection, BMI is significantly associated with tumour growth (*P* value = 0.09). The effects of body size on tumour growth and time to symptomatic detection work in the same direction, increasing the size of the tumour, and BMI and TBA are strongly correlated variables. It is quite possible that with a somewhat larger sample size we would have sufficient power to show that body size is significantly associated, separately, with both tumour growth and time to symptomatic detection. However, the estimated direction of the effect for BMI on tumour growth is as we would expect, and other studies have already found significant associations between BMI and Ki-67 among women older than 40 years [8].

We obtained somewhat stronger evidence that, when including BMI in the model for tumour growth, breast size affects symptomatic detection (*P* value = 0.02). Since our study is the first to test such an association, it is not possible to make a direct comparison with other studies. However, there is some indirect support for our result in other studies. Using a multivariate logistic regression analysis, Boyd et al. [23] found that high BMI was more common in screening cases than in interval cases, when taking the variable mammographic density into account. This is in line with BMI (through its positive association with breast size) delaying symptomatic detection. However, low mammographic density has the same effect of increasing the probability of being screen-detected. In the data set used in this article, tumour size was more strongly associated with TBA than with BMI (Table 2).

Women with high BMI have higher estrogen levels than lean women and this might increase the growth of a tumour. However, women receiving hormone replacement therapy (HRT) also have high estrogen levels because of this exogenous source of estrogens. Therefore, it has been suggested that any relationship between tumour progression and BMI could be weaker among women receiving HRT [24]. A more appropriate tumour growth model may be one with a main effect for HRT use and an interaction effect between HRT use and BMI.

In future studies, we aim to extend the model to include more breast cancer risk factors/covariates. For example, some measure of HRT could be included in the tumour growth model. It has also been discussed whether mammographic density affects tumour growth, although, to date, little evidence has been presented for this association [25]. It may also be of value to quantify the role of age in tumour growth (although age at diagnosis was found not to be associated with tumour size, among postmenopausal women, in a basic regression analysis). However, the estimation procedure is computationally expensive and (as reported in our earlier study [12]) computational time, especially for variance estimates, increases when adding extra covariates.

One of the main reasons for developing the types of models described here is to understand more about the relationships between risk factors and the biology of breast cancer. As Vilaprinyo et al. [26] point out, such information will be needed to make screening more efficient. Although breast cancer mammography screening programmes are widely used and often age-based, the efficiency of screening programmes is still debated, and it has been argued that individualised screening programmes are needed to reduce overdiagnosis and overtreatment [27]. Based on simulation studies, researchers are testing different individualised screening strategies [26]. Although Vilaprinyo et al. are optimistic about their results, they also indicate that, in order to make screening more efficient, more information about how to identify women who would benefit most from screening is needed. The optimal screening frequency will depend on the cancer growth rate, with screening being most suitable for slow-growing precancerous tumours [28]. The sensitivity of a screening procedure and factors affecting it are also important for deciding which type of screening a woman should receive [29]. Within the debate on personalised screening, factors which could delay symptomatic detection have not been discussed to the same extent. However, they may be important. We have shown that breast size significantly delays symptomatic detection. This knowledge can potentially be used when planning individualised screening.

## Conclusions

Sophisticated tumour growth modelling is needed to unravel the mechanisms behind the association between a large body size and larger breast cancer tumours at diagnosis. We develop a novel continuous tumour growth model with submodels for tumour growth, time to symptomatic detection, and screening sensitivity and further describe approaches for including covariates in these submodels. Using our growth model, we found that a large breast size is associated with a delayed symptomatic detection in postmenopausal women.

## Appendix - statistical methods

We begin this section by describing the three submodels which we postulate and which in turn are used to calculate the probability for a detected tumour to be of a specific size (at time of diagnosis), conditional on screening history and whether the tumour was detected through screening or symptomatically.

### Model for tumour growth

In the first submodel, for tumour growth, tumours are assumed to be spherical and to grow exponentially with a constant volume doubling time. The volume (in cubic millimetres) for a tumour, at time *t*, is specified as

1 $$ V(t)={V}_{cell}{e}^{t/r}, $$

where *t* is the time in years after tumour onset, *V*_*cell*_ is the volume of one cell, and *r* is the inverse growth rate. To allow for tumours to grow at different rates, the inverse growth rate, *r*, is modelled as a random variable, *R*, which is assumed to follow a gamma distribution, with shape parameter *τ*_1_ and rate parameter *τ*_2_. The density function for *R* is

2 $$ {f}_R(r)=\frac{{\tau_2}^{\tau_1}}{\Gamma \left({\tau}_1\right)}{r}^{\tau_1-1}{e}^{-{\tau}_2r},r\ \ge\ 0, $$

and *E*(*R*) = *τ*_1_/*τ*_2_. This model, which has been chosen partly because of mathematical tractability, has also been used by Bartoszyński et al. [17] and Plevritis et al. [18]. Neither of those articles allowed growth rates to vary according to an observed factor/covariate. In Abrahamsson and Humphreys [12], we estimated *τ*_1_ and *τ*_2_. Here, we adapt the model in order to incorporate BMI as a covariate. We model dependence of growth rates on BMI by using a log-linear scale of the mean. We use a mean reparametrisation such that *E*(*R*) = *τ*_1_/*τ*_2_ = *μ* and *V*(*R*) = *τ*_1_/*τ*_2_^2^ = *σ* ^2^ *μ* ^2^, where *σ* is the constant coefficient of variation, and specify

3 $$ \log \left(\mu \right)={\alpha}_1+{\alpha}_2b, $$

where *b* = *BMI* (centered on the mean value in the study). The parameter *α*_2_ summarises the association between BMI and tumour growth. With this extension of the model, any covariate can be added to the tumour growth model in future studies.

### Model for symptomatic detection

We assume that time to symptomatic detection, *T*_*det*_, counted from the time that the tumour consisted of one cell, depends on the size of the tumour through the following hazard function

4 $$ P\left({T}_{det}\ \in\ \left[t,t+dt\right)\Big|{T}_{det}>t\right)=\lambda V(t)dt+o(dt). $$

This model is also used by Bartoszyński et al. [17] and Plevritis et al. [18]. We make the important extension to the model, to allow time to symptomatic detection to depend on breast size (or any other covariate in future studies), by writing

5 $$ \log \left(\uplambda \right)={\eta}_1+{\eta}_2s, $$

where *s* represents breast size as measured by the standardised TBA (centered on the mean value and divided by the standard deviation in the study) calculated from a mammogram. Inference on *η*_2_ provides information on the relationship between the size of a woman’s breast and time to symptomatic detection (caused by difficulties in palpation).

### Model for screening sensitivity

Larger tumours are easier to find through screening than small ones, and tumours can also be masked in breasts with high mammographic density. We therefore assume mammography screening sensitivity to be a function of tumour size and mammographic density. Its functional form (logistic) is adopted from Weedon-Fekjær et al. [19] and is written as

6 $$ S\left(d,m\right)=\frac{ \exp \left({\beta}_1+{\beta}_2d+{\beta}_3m\right)}{1+ \exp \left({\beta}_1+{\beta}_2d+{\beta}_3m\right)}, $$

where *S*(*d*, *m*) represents the probability that, from a mammographic image, a radiologist will detect a tumour of size *d* in a breast with PMD *m* (0 ≤ *m* ≤ 1). The size of a tumour, *d*, is measured in terms of its diameter (in millimetres).

### Likelihood function

We model the size of a woman’s tumour conditioning explicitly on her screening history and how her tumour was detected. We treat the diameter of the tumours as coming from separate multinomial distributions, consisting of a number of tumour size intervals. The tumour size distributions are continuous in nature, and the multinomial distributions are used as approximations. We evaluate and maximise the likelihood

7 $$ L\left(\mathbf{o}\Big|\boldsymbol{\uptheta} \right)=\prod_j\prod_i{p_{i,j}}^{o_{i,j}}, $$

where *p*_*i*,*j*_ is the (conditional) probability for a tumour in woman *j* to be detected in size interval *i*. In (7), *o*_*i*,*j*_ equals 1 if woman *j* has a tumour detected in size interval *i*, and 0 otherwise, and ***o*** is a matrix consisting of all values *o*_*i*,*j*_. Furthermore, ***θ*** is a vector of eight parameters whose values we estimate by maximising the likelihood function, (7).

The probability for a woman’s tumour to be detected in a specific size interval (*p*_*i*,*j*_) is specified to be dependent on body size covariates and PMD as well as the dates of her previous negative screenings and the detection mode of her tumour. We calculate the value of *p*_*i*,*j*_ after first postulating the three submodels (as functions of body size covariates and PMD).

For screening cases, *p*_*i*,*j*_ can be shown to be

8 $$ {p}_{i,j}\propto S\left(i,m\right){\displaystyle \sum_f}{\displaystyle \sum_g}P\left(\begin{array}{c}\hfill Tumour\  is\ \hfill \\ {}\hfill\ of\  size\ i\  at\ \hfill \\ {}\hfill screening\hfill \end{array}\left|\begin{array}{c}\hfill In\  the\  absence\  of\  screening,\hfill \\ {}\hfill the\  tumour\  will\  be\  detected\hfill \\ {}\hfill at\  size\ g,\ by\  symptoms,\hfill \\ {}\hfill f\  months\  later\hfill \end{array}\right.\right)\times $$

9 $$ P\left( The\; tumour\; is\; symptomatically\kern0.20em  detected\; of\; size\;g\right)\times $$

10 $$ {\displaystyle \sum_s}P\left(\begin{array}{c}\hfill Tumour\  is\  not\  detected\hfill \\ {}\hfill at\  earlier\  screening s\hfill \end{array}\left|\begin{array}{c}\hfill Tumour\  is\  of\  size\ i\  at\  latest\  screening\hfill \\ {}\hfill and\  of\  size\ s\  at\  the\  screening\  before\  that\hfill \end{array}\right.\right)\times $$

11 $$ P\left(\left.\begin{array}{c}\hfill Tumour\  is\  of\  size\ s\  at\  the\hfill \\ {}\hfill second\  latest\  screening\hfill \end{array}\right| Tumour\  is\  of\  size\ i\  at\  latest\  screening\right), $$

where rows (10) and (11) are omitted for women with no negative screening. See Abrahamsson and Humphreys [12] for the derivation of this formula. On row (8), *S*(*i*, *m*) is evaluated by using function (6) and the midpoint of the size interval *i*. The conditional probability in the summation over *f* and *g* on row (8) is evaluated by back-calculation (tracking the tumour backwards in time from its hypothetical symptomatic detection as if the woman had never attended screening) by using the conditional cumulative density function for the growth rate conditioning on the size at symptomatic detection (using equation (10) in Abrahamsson and Humphreys [12]). The probability on row (9) is evaluated by using the cumulative density function for the growth rate (equation (4) in [12]). The screening sensitivity function (6) is used to calculate the probability on row (10) for the earlier negative screenings. Finally, the probability on row (11) is evaluated in a similar way to row (8) [12].

For symptomatic cases, *p*_*i*,*j*_ can be shown to be

12 $$ {p}_{i,j}\propto P\left( The\  tumour\  is\  symptomatically\  detected\  at\  size\ i\right)\times $$

13 $$ {\displaystyle \sum_s}P\left(\begin{array}{c}\hfill Tumour\  is\  not\  detected\hfill \\ {}\hfill at\  earlier\  screening s\hfill \end{array}\left|\begin{array}{c}\hfill Tumour\  is\  of\  size\ i\  at\  symptomatic\  detection\hfill \\ {}\hfill and\  of\  size\ s\  at\  last\  screening\hfill \end{array}\right.\right)\times $$

14 $$ P\left(\begin{array}{c}\hfill Tumour\  is\  of\  size\ s\  at\ \hfill \\ {}\hfill last\  screening\hfill \end{array}\left|\begin{array}{c}\hfill Tumour\  is\  of\  size\ i\  at\hfill \\ {}\hfill symptomatic\  detection\hfill \end{array}\right.\right), $$

where rows (13) and (14) are omitted for women with no screening history. In the above formula, rows (12), (13), and (14) are, respectively, similar to (9), (10), and (8).

## Abbreviations

BMI: Body mass index
HRT: Hormone replacement therapy
PMD: Percentage mammographic density
TBA: Total breast area
